# Elevated expression of G3BP1 associates with YB1 and p‐AKT and predicts poor prognosis in nonsmall cell lung cancer patients after surgical resection

**DOI:** 10.1002/cam4.2579

**Published:** 2019-09-27

**Authors:** Hongmei Zheng, Yuting Zhan, Yuting Zhang, Sile Liu, Junmi Lu, Yang yang, Qiuyuan Wen, Songqing Fan

**Affiliations:** ^1^ Department of Pathology The Second Xiangya Hospital Central South University Changsha Hunan China

**Keywords:** biomarker, G3BP1, nonsmall cell lung cancer, p‐AKT, prognosis, YB1

## Abstract

**Purpose:**

G3BP1 is an RNA‐binding protein and plays roles in regulating signaling pathway. YB‐1 is a DNA/RNA binding protein encoded by *YBX1* gene. Phosphorylated AKT (p‐AKT) acts as a pivotal molecule in PI3K/AKT pathway. YB‐1 drives stress granules (SGs) formation by activating G3BP1 translation under diverse conditions. SGs are involved in many different metabolic and signaling pathways which may include PI3K/AKT/mTOR. So far, there has been no report on the relationship between expression of G3BP1, p‐AKT, and YB1 and clinicopathological features/prognosis in surgically resected nonsmall cell lung cancer (NSCLC) patients.

**Methods:**

In this study, data from TCGA (The Cancer Genome Atlas) were downloaded to investigate the mRNA expression of G3BP1 and YB1 (YBX1) and their correlation in NSCLC. Also, expression of G3BP1, YB1, and p‐AKT proteins was studied using immunohistochemistry in tissue microarrays of NSCLC and in noncancerous lung tissues.

**Results:**

We found that the mRNA expression of G3BP1 and YB1 was higher in NSCLC tissues (both *P < *.05), and G3BP1 was positively correlated with YB1 in mRNA level (*r* = .399, *P* < .001). Also, expression of G3BP1, YB1, and p‐AKT proteins was higher in NSCLC tissues (all *P* < .05). And higher expression of G3BP1 and YB1 proteins was seen in patients with clinical stage II and III compared with stage I (both *P* < .05). Besides, expression of G3BP1 protein had a positive correlation with YB1 and p‐AKT (both *P* < .05). Moreover, overall survival was shorter in patients with overexpression of G3BP1, YB1, and p‐AKT proteins (all *P* < .05). Multivariate analysis confirmed that overexpression of G3BP1 protein was an independent poorer prognostic factor for NSCLC patients (*P* = .039).

**Conclusion:**

G3BP1 may play a crucial role in activating PI3K/AKT/mTOR pathway. G3BP1 might be served as a novel prognostic biomarker for surgically resected NSCLC patients, which afforded new insights into the study on the mechanism and therapy of NSCLC.

## Graphical abstract

The overexpression of G3BP1, YB1, and p‐AKT protein was associated with clinicopathological features in NSCLC and the expression of G3BP1 had a positive correlation with YB1 and p‐AKT. G3BP1 may play a crucial role in activating PI3K/AKT/mTOR signaling pathway. G3BP1 protein might be served as a novel prognostic biomarker for NSCLC patients with surgical resection.

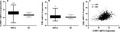

## 1 INTRODUCTION

Lung cancer originates from the alveoli and bronchial mucosal epithelium, and is one of the malignant tumors with the highest incidence and mortality in China and the world, which seriously endangers human health.^1^, ^2^ Based on the WHO criteria, lung cancer is usually divided into nonsmall cell lung cancer (NSCLC) and small cell lung cancer (SCLC), of which NSCLC accounts for more than 80%.^3^ Early lung cancer is mostly asymptomatic, and most patients are in advanced stage at the first diagnosis, resulting in a 5‐year survival rate of only about 16% for lung cancer.^4^ The occurrence and development of lung cancer is a complex process mediated by multiple genes and signaling pathways. Therefore, the further study of molecular biomarkers for lung cancer has far‐reaching significance for timely understanding its malignant degree, predicting metastasis, judging prognosis and finding some new therapeutic targets.

Ras‐GTase‐activating protein SH3 domain binding protein 1 (G3BP1) is the first RasGAP SH3 domain binding protein isolated through immunoprecipitation by Parker et al,^5^ which plays a key role in regulating Ras signal transduction and belongs to RNA‐binding protein family.^6^ Its dephosphorylation facilitates the assembly of stress granules (SGs) which maximize the ability of cells to survive to repair stress‐induced changes under stress conditions.^7^, ^8^ Y‐box binding protein 1 (YB‐1), an RNA/DNA binding protein, is ubiquitous in prokaryotic and eukaryotic cells, which contains a highly conserved cold shock domain and can specifically bind to the Y‐box sequence in the enhancer and promoter of the target gene.^9^ Moreover, YB1, as a transcription and translation factor, plays the vital role in regulating cell proliferation, differentiation and stress response.^10^ Akt, known as protein kinase B (PKB), is a highly conserved threonine/serine protein kinase in evolution. It mainly exists in the cytoplasm and acts as a pivotal molecule in activating PI3K/AKT signaling pathway.^11^ Phosphorylated protein kinase B (p‐AKT) can regulate many proteins related to cell metabolism, apoptosis, proliferation and differentiation, thus inhibiting cell apoptosis and promoting the growth of cancer cells.^12^ It was reported that YB‐1 could bind to the 5′UTR of G3BP1 transcripts to up‐regulate translation of G3BP1, thus controlling the validity of the G3BP1 SG nucleator in SG assembly and promoting the tumor progression.^13^

As far as we know, there has been no report on whether the expression of G3BP1, p‐AKT, and YB1 proteins is related to clinicopathological features of NSCLC patients with surgical resection. And the relationship between G3BP1, YB1, and p‐AKT in NSCLC remains unclear. In the current study, we detected the expression of G3BP1, p‐AKT, and YB1 proteins in 48 cases of noncancerous control lung tissues and 247 cases of NSCLC by IHC and investigate the mRNA level of G3BP1 and YB1, and thereby exploring the relationship between the expression of G3BP1, YB1, and p‐AKT proteins and clinicopathological features and their prognostic significance in NSCLC.

## 2 MATERIALS AND METHODS

### 2.1 TCGA database

The TCGA database was downloaded to analyze the mRNA expression of G3BP1 and YB1 in NSCLC tissues, and to further examine their correlation. There are 1017 cases of NSCLC tissues and 110 NC (normal control) tissues.

### 2.2 Ethical statement

All protocols were confirmed by The Second Xiangya Hospital of Central South University Ethics Review Board (Scientific and Research Ethics Committee, No. S039/2011) and all research samples were collected with informed consent. If the patient is juvenile, a written consent will be signed by guardian on behalf of the juvenile participating in this study.

### 2.3 Patient cohorts and tissue microarrays (TMAs)

In this study, 247 cases of NSCLC postoperative specimens were selected randomly from The Second Xiangya Hospital of Central South University (Changsha, China), including ADC (n = 127), SCC (n = 120) and noncancerous lung tissues (n = 48) during the period from 2002 to 2011. All patients had complete clinical and follow‐up data. Overall survival time was defined as the time from initial diagnosis to death or last follow‐up. None of patients had previously been treated with preoperative treatment, but all had received chemotherapy within 30 days after surgery. There were 183 males and 64 females with an average age of (56.0 ± 8.8) years, 72 cases of clinical stage I and 175 cases with stage II and III. At the end of the follow‐up, 93 patients died and 154 survived. We chose the cutoff of age (<56, ≥56) on the basis of these patients’ average age. The staging classification of each case was carried out with the Eighth Edition Lung Cancer Stage Classification and all patients had a clear pathological diagnosis based on the WHO histological classification.^14^ Tissue microarrays were made based on the technology previously described.^15^

### 2.4 IHC and scores

The G3BP1, YB1, and p‐AKT proteins were stained by ready‐to‐use Envision TM^+^ Dual Link Systenm‐HRP methods (Dako). As mentioned in previous,^16^, ^17^ the staining condition of each antibody was followed based on laboratory experience. A 1:300 dilution of primary antibody to G3BP1 (Monoclonal Mouse antibody, Catalog: sc‐365338, Santa Cruz Biotechnology), a 1:200 dilution of primary antibody to YB1 (Monoclonal Rabbit antibody, Catalog: #4202, Cell Signaling Technology) and a 1:200 dilution of the primary antibody to p‐AKT (Rabbit polyclonal antibody, Catalog Ab81283, Abcam) were applied to measure expression of these three proteins. The specificity of the antibody was confirmed by matched IgG isotype antibody as the negative control. Each experiment included positive control slide.

The IHC staining was independently evaluated by SF and QW at 200× magnification light microscopy, who did not know the clinicopathological data. The evaluation was based on a semi‐quantitative method described as follows 16: total score = intensity score × percentage score. The staining intensity of G3BP1, YB1, and p‐AKT was scored as 3 (strong), 2 (moderate), 1 (weak), and 0 (negative), besides, staining percentage was scored as 4 (76%‐100%), 3 (51%‐75%), 2 (26%‐50%), 1 (1%‐25%), and 0 (0%). The scores of G3BP1, YB1, and p‐AKT proteins ranged from 0 to 12, and the optimal cut‐off levels were 6, 4, and 2, based on the OS of NSCLC patients using the log‐rank test. G3BP1, YB1, and p‐AKT were divided into low expression and high expression. All differences in scores were resolved through discussion, with 95% consistency between the two evaluators.

### 2.5 Statistical analysis

All data management and statistical analyses were performed by SPSS 24.0. Unpaired *t* test was used to examine the mRNA expression of G3BP1 and YB1 in NSCLC and normal control tissues. Chi‐square test was used to analyze the association between the expression of G3BP1, p‐AKT, and YB1 proteins and clinicopathological features of NSCLC. The correlation among G3BP1, p‐AKT, and YB1 was evaluated by Spearman's rank correlation coefficient. The survival rate was performed with the Kaplan‐Meier analysis, and the two survival rate curves were compared by log‐rank test. Cox comparative hazards model was used to evaluate independent prognostic factors. *P* < .05 (two‐sided) was considered to be statistically significant.

## 3 RESULTS

### 3.1 Bioinformatics analysis of G3BP1 and YB1 in NSCLC

**Figure 1:**
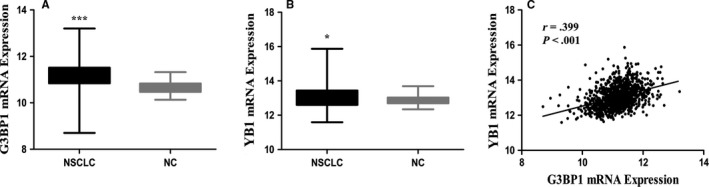
Bioinformatics analysis of G3BP1 and YB1 in NSCLC. mRNA expression of G3BP1 (A) and YB1 (B) was significantly higher in NSCLC tissues than that in NC (normal control) tissues (*P* < .0001 and *P* = .017, respectively). G3BP1 (C) was positively correlated with YB1 in mRNA level (*r* = .399, *P* < .001)

A total of 1017 NSCLC tissues and 110 normal control tissues were selected from the TCGA database. The results showed that the mRNA expression of G3BP1 and YB1 was significantly higher in NSCLC tissues than that in normal control tissues (both *P* < .05). Besides, we also found that G3BP1 was positively correlated with YB1 in mRNA level (*r* = .399, *P* < .001; Figure 1).

### 3.2 Elevated expression of G3BP1, p‐AKT, and YB1 proteins was evidently higher in NSCLC

**Figure 2:**
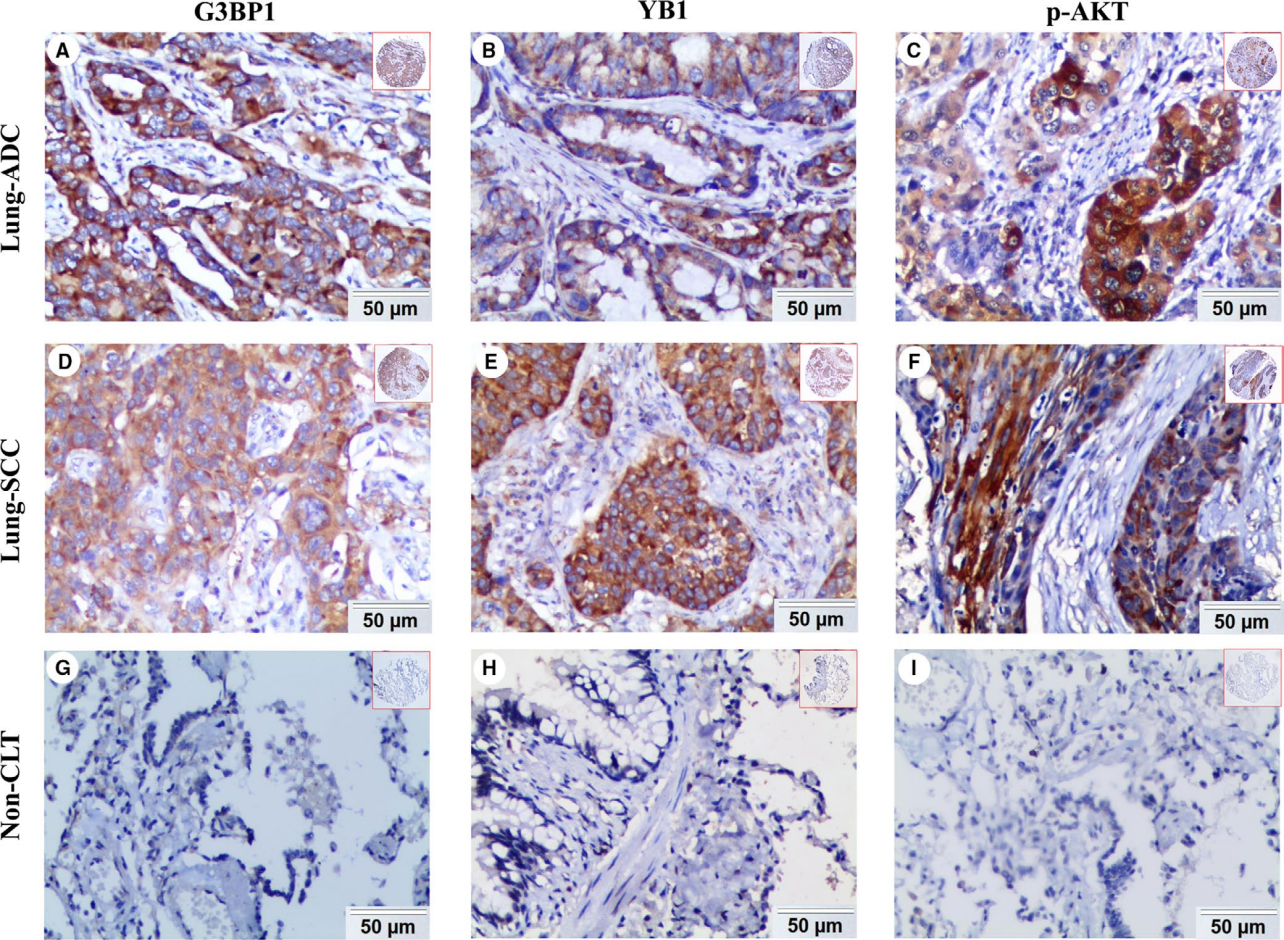
Expression of G3BP1, YB1, and p‐AKT proteins in lung ADC, lung SCC and Non‐CLT (noncancerous lung tissues) was detected by IHC. High expression of G3BP1 (A), YB1 (B), and p‐AKT (C) proteins was showed in lung ADC. High expression of G3BP1 (D), YB1 (E), and p‐AKT (F) proteins was also showed in lung SCC. Negative staining of G3BP1 (G), YB1 (H) and p‐AKT (I) proteins was found in noncancerous lung tissue. (IHC, DAB staining, original magnification 200× and 40×)

We detected the expression and subcellular location of G3BP1, p‐AKT and YB1 proteins in noncancerous lung tissues and NSCLC tissues. The positive expression of G3BP1, YB1, and p‐AKT proteins was discovered in the cytoplasm and membrane of cancer cells, whereas nuclear staining was rarely identified (Figure 2A‐F). There was no positive staining of G3BP1, YB1, and p‐AKT proteins in noncancerous lung tissues (Figure 2G‐I).

**Figure 3:**
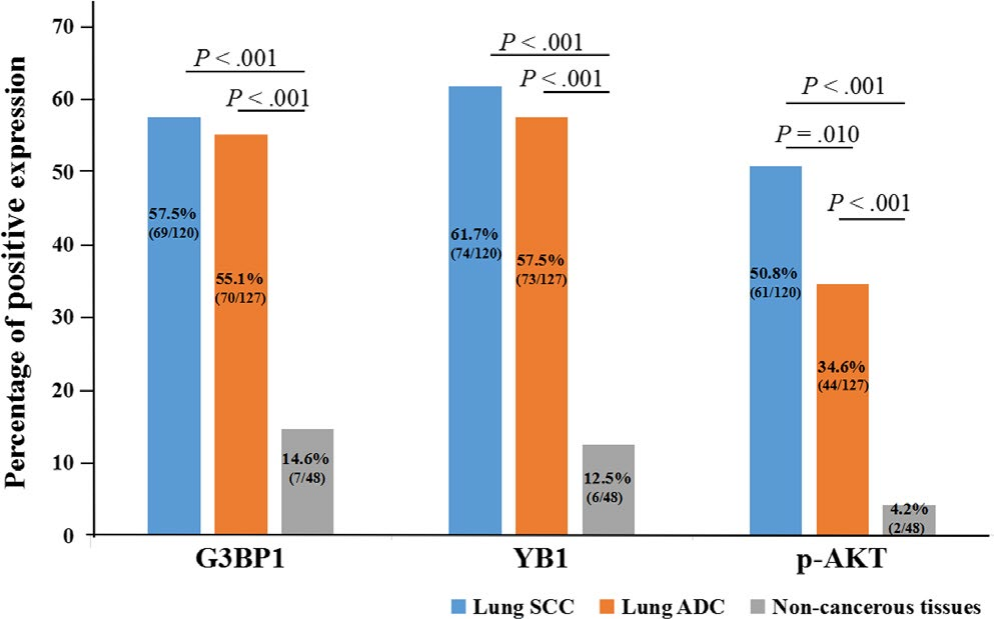
The comparison of expression of G3BP1, YB1, and p‐AKT in lung SCC and lung ADC compared to the noncancerous lung tissues. The expression of G3BP1, YB1, and p‐AKT proteins in lung SCC and lung ADC was significantly higher than those in noncancerous lung tissues (all *P* < .001). Besides, the expression of p‐AKT was higher in lung SCC than lung ADC, which was significant differences (*P = *.010)

The positive percentage of increased expression of G3BP1 and YB1 proteins was 57.5% (69/120) and 61.7% (74/120) in lung SCC, 55.1% (70/127) and 57.5% (73/127) in lung ADC, and 14.6% (7/48) and 12.5% (6/48) in noncancerous control lung tissues, respectively. In terms of p‐AKT, the positive percentage of high expression was 4.2% (2/48), 50.8% (61/120), 34.6% (44/127) and in noncancerous lung tissues, lung SCC and lung ADC. As shown in Figure 3, the elevated expression of G3BP1, YB1, and p‐AKT proteins was significantly higher in lung ADC and SCC tissues (all *P* < .001). There was also a significant difference in the expression of p‐AKT protein between lung SCC and ADC (*P* = .010).

### 3.3 Relationship between clinicopathological features and high expression of G3BP1, p‐AKT, and YB1 proteins in NSCLC

We further studied the relationship between clinicopathological parameters, including gender, age, histological type, clinical stage, pathological degree and LNM (lymph node metastasis) status and overexpression of G3BP1, YB1, and p‐AKT proteins by chi‐square test. The data in Table 1 revealed high expression of G3BP1 and YB1 proteins was positively correlated with the clinical stage (*P* = .016 and *P* = .025). But there was no relationship in terms of gender, age, pathological degree, and histological type (all *P* > .05). NSCLC patients with LNM had the higher expression of G3BP1 than those without LNM, but the statistical significance was weak (*P* = .054). As for p‐AKT, lung SCC patients had evidently increased expression than lung ADC patients (*P = *.010), but there was no statistical difference in other clinical variables (all *P > *.05).

**Table 1:** Association between expression of G3BP1, YB1, and p‐AKT proteins and clinicopathological features of NSCLC (n = 247)

Clinicopathological features	G3BP1		*P*	YB1		*P*	p‐AKT		*P*
	H (%)	L (%)		H (%)	L (%)		H (%)	L (%)	
Age (y)									
<56 (n = 108)	62 (57.4)	46 (42.6)	.752	63 (58.3)	45 (41.7)	.739	41 (38.0)	67 (62.0)	.203
≥56 (n = 139)	77 (55.4)	62 (44.6)		84 (60.4)	55 (39.6)		64 (46.0)	75 (54.0)	
Gender									
Female (n = 64)	30 (46.9)	34 (53.1)	.078	32 (50.0)	32 (50.0)	.072	23 (35.9)	41 (64.1)	.217
Male (n = 183)	109 (59.6)	74 (40.4)		115 (62.8)	68 (37.2)		82 (44.8)	101 (55.2)	
Histological type									
ADC (n = 127)	70 (55.1)	57 (44.9)	.706	73 (57.5)	54 (42.5)	.503	44 (34.6)	83 (65.4)	.010*, *
SCC (n = 120)	69 (57.5)	51 (42.5)		74 (61.7)	46 (38.3)		61 (50.8)	59 (49.2)	
Pathological degree									
Well/moderated differentiation (n = 113)	65 (57.5)	48(42.5)	.717	62 (54.9)	51 (45.1)	.172	42 (37.2)	71 (62.8)	.119
Poor differentiation (n = 134)	74 (55.2)	60 (44.8)		85 (63.4)	49 (36.6)		63 (47.0)	71 (53.0)	
Clinical stages									
Stage I (n = 72)	32 (44.4)	40 (55.6)	.016*, *	35 (48.6)	37 (51.4)	.025*, *	33 (45.8)	39 (54.2)	.498
Stages II and III (n = 175)	107 (61.1)	68 (38.9)		112 (64.0)	63 (36.0)		72 (41.1)	103 (58.9)	
LNM status									
LNM (n = 136)	84 (61.8)	52 (38.2)	.054	82 (60.3)	54 (39.7)	.782	56 (41.2)	80 (58.8)	.639
No LNM (n = 111)	55 (49.5)	56 (50.5)		65 (58.6)	46 (41.4)		49 (44.1)	62 (55.9)	

Note

The average age of all patients with NSCLC was 56.0 ± 8.78 years.

Abbreviations: ADC, adenocarcinoma; LNM, lymph node metastasis; SCC, squamous cell carcinoma.

* *P* < .05.

### 3.4 The correlation between expression of G3BP1, p‐AKT, and YB1 proteins in NSCLC

The correlation between increased expression of G3BP1, p‐AKT, and YB1 proteins in NSCLC was revealed in Table 2. Data indicate that the high expression of G3BP1 protein positively correlated with the expression of YB1 and p‐AKT proteins (*r* = .320, *P* < .001; *r* = .213, *P* = .001, respectively). But there was no correlation between YB1 and p‐AKT in NSCLC (*r* = .075, *P* = .239). These data suggested that aberrant high expression of G3BP1 might play a key role in p‐AKT signaling pathway and promoting cell survival in NSCLC.

**Table 2:** The pairwise association between expression of G3BP1, YB1, and p‐AKT proteins in the 247 cases of NSCLC

	G3BP1	YB1	p‐AKT
G3BP1			
Spearman's correlation coefficient	1	0.320	0.213
Sig. (2‐tailed)		0.000*, *	0.001*, *
YB1			
Spearman's correlation coefficient	0.320	1	0.075
Sig. (2‐tailed)	0.000*, *		0.239
p‐AKT			
Spearman's correlation coefficient	0.213	0.075	1
Sig. (2‐tailed)	0.001*, *	0.239	

* *P* < .05.

### 3.5 Effects of high expression of G3BP1, p‐AKT, and YB1 proteins on the survival time of NSCLC patients

**Figure 4:**
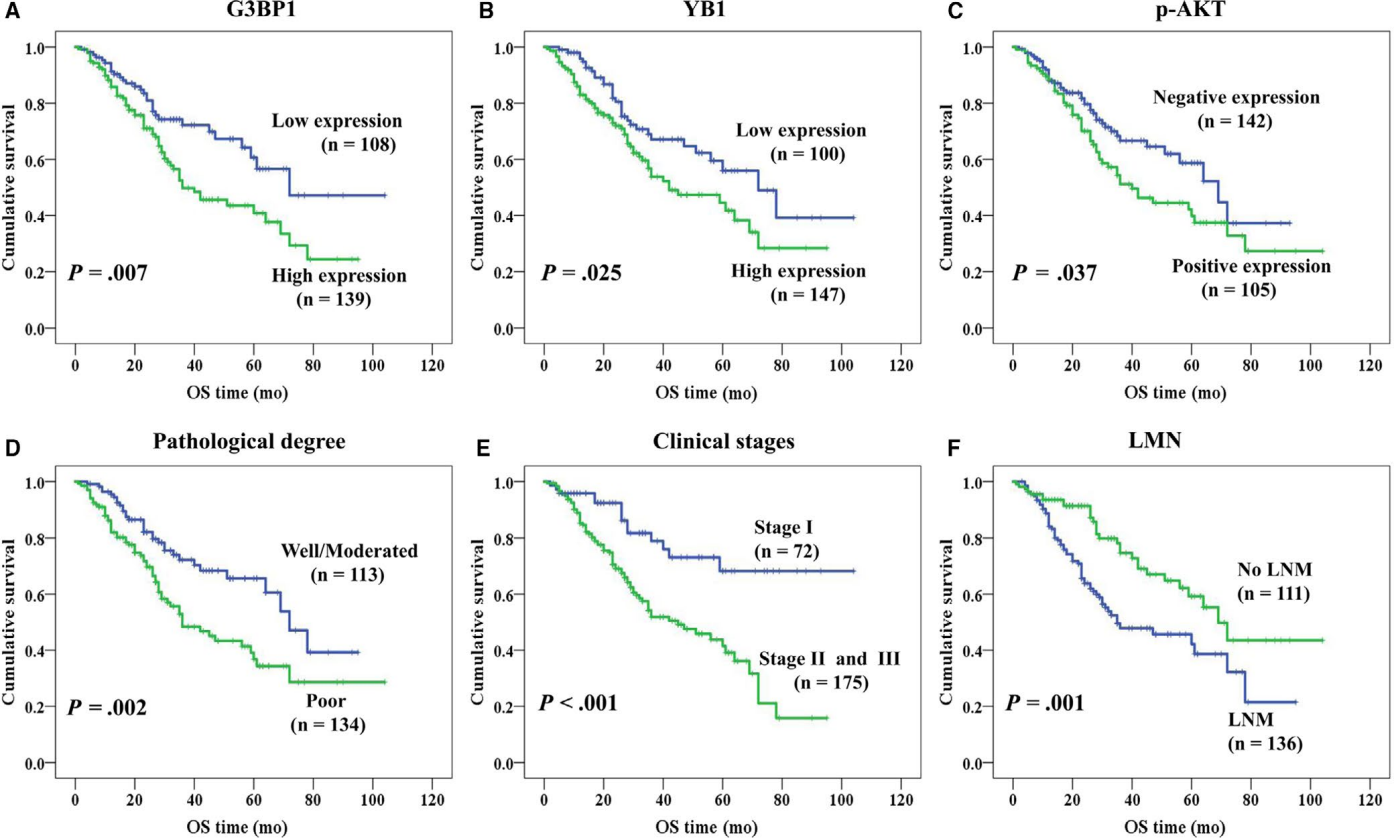
Kaplan‐Meier curves for overall survival of NSCLC patients assessed using the log‐rank test (all tests were two‐sided). Patients with high expression of G3BP1 (A, *P* = .007) and YB1 (B, *P* = .025) had longer survival time. Low expression of p‐AKT was significantly related to better prognosis (C, *P* = .037), as well as well/moderated differentiation (D, *P* = .002), stage I (E, *P < *.001) and without LNM (F, *P* = .001)

In univariate survival analysis, we used Kaplan‐Meier survival curves to analyze the relationship between OS rate and clinicopathological features, G3BP1, YB1, and p‐AKT protein and also used the log‐rank test to evaluate statistical significance. The cumulative survival rate was 95.5% at 6 months, 88.2% at 1 year, 59.4% at 3 years, and 49.2% at 5 years. NSCLC patients with low expression of G3BP1 (Figure 4A, *P* = .007), YB1 (Figure 4B, *P = *.025) and p‐AKT protein (Figure 4C, *P* = .037) survived longer than those with high expression. Compared with NSCLC patients with poor differentiation, higher OS rate could be seen for those with well and moderated differentiation (Figure 4D, *P* = .002), whereas, the OS rate was lower for patients with stage II and III than those with stage I (Figure 4E, *P* < .001). Interestingly, we also found that patients without LNM survived longer than those with LNM (Figure 4F, *P* = .001).

Cox proportional hazard regression analysis was used to further verify whether the overexpression of G3BP1, p‐AKT, and YB1 proteins was the independent prognostic factors in NSCLC patients, which was shown in Table 3. The results indicated that high expression of G3BP1 was proved to be a poor prognostic factor for NSCLC patients (*P *= .039), as well as pathological degree (*P = *.002) and clinical stage (*P* = .036). Nevertheless, increased expression of p‐AKT and YB1 had no significant impact on prognosis of NSCLC patients. Besides, no prognostic significance was detected with histological type, LNM status, age, and gender in NSCLC patients (all *P > *.05).

**Table 3:** Summary of univariate analysis and multivariate analysis for overall survival in 247 cases of NSCLC patients

Variables	Univariate analysis			Multivariate analysis		
	Average survival time (SE)	95%CI	*P*	Exp (B)	95.0% CI	*P*
G3BP1						
High expression	49.84 (3.57)	42.85‐56.83	.007*	0.607	0.378‐0.975	.039*
Low expression	69.68 (5.20)	59.49‐79.89				
YB1						
High expression	51.32 (3.72)	44.03‐58.62	.025*	0.882	0.556‐1.399	.594
Low expression	66.77 (5.05)	56.83‐76.68				
p‐AKT						
Positive expression	53.09 (4.42)	44.44‐61.75	.037*	0.694	0.447‐1.077	.103
Negative expression	60.13 (4.02)	52.25‐68.00				
Clinical stages						
Stage I	80.78 (5.35)	70.30‐91.27	.000*	0.486	0.248‐0.956	.036*
Stage II and III	48.21 (3.13)	42.09‐54.33				
LNM status						
LNM	48.91 (3.81)	41.44‐56.37	.001*	1.457	0.862‐2.463	.160
No LNM	69.36 (4.82)	59.91‐78.81				
Pathological degree						
Well/moderated	64.85 (4.11)	56.79‐72.92	.002*	0.493	0.316‐0.770	.002*
Poor	51.87 (4.29)	43.46‐60.27				
Histological type						
ADC	51.80 (3.48)	44.97‐58.62	.416	1.510	0.945‐2.413	.085
SCC	65.86 (4.84)	56.37‐75.34				
Gender						
Female	60.49 (4.94)	50.81‐70.17	.218	0.698	0.415‐1.173	.175
Male	56.94 (3.95)	49.20‐64.67				
Age						
<56	60.82 (4.48)	52.05‐69.59	.851	0.900	0.587‐1.381	.631
≥56	54.29 (3.59)	47.26‐61.33				

Abbreviations: ADC, adenocarcinoma; CI, confidence interval; Exp(β), odds ratio; LNM, lymph node metastasis; SCC, squamous cell carcinoma; SE, standard error.

* *P* < .05.

## 4 DISCUSSION

Stress granules (SGs) are formed by cytoplasmic RNA particles when exposed to some environmental stresses, such as hypoxia, heat shock, and arsenite, which contain several RNA‐binding proteins, such as eukaryotic initiation factor (eIF)3, G3BP1, eIF4G and several ribosomal proteins and have antioxidant and anti‐apoptotic effects.^18^ G3BP1 plays important roles in the formation of SGs, which has proved that the knockdown of G3BP1 reduces the assembly of SGs, thus making tumor cells have chemotherapeutic resistance and survival advantage.^19^, ^20^, ^21^ G3BP1 also inhibits apoptosis by regulating Src/FAK, TGF‐β/Smad, Ras, and p53 signaling pathways and thereby promotes tumor cell proliferation and metastasis.^22^ Compared with normal tissues or cells, G3BP1 was confirmed to be overexpressed in many malignant tumors. Zhang et al^23^ revealed that G3BP was highly expressed in esophageal squamous carcinoma and was closely associated with the lymph node metastasis and survival. Besides, it was reported that G3BP1 promoted the metastasis of hepatocellular carcinoma through upregulation of Slug expression and might be a new predictor for prognosis.^24^ Also, G3BP1 was proved to be positively related to tumor classification, tumor size, lymph node metastasis, and TNM stage and reduced overall survival in gastric cancer patients.^25^ Intriguingly, Barnes et al^26^ found that overexpression of G3BP1 protein in breast cancer might be involved in the HER2 signaling pathway. In addition, it was found that PTEN could down‐regulate the expression of G3BP by inhibiting PI3K/AKT signaling pathway in normal cells and there was a negative correlation between the expression of PTEN and G3BP.^27^ In this work, we showed G3BP1 was highly overexpressed in NSCLC tissues including lung ADC and SCC, compared with noncancerous lung tissues, which was consistent with the above findings and we further confirmed that it had an evidently positive relation with clinical stage of NSCLC patients. What's more, our result indicated that high expression of G3BP1 protein was identified as an independent poorer prognostic factor for patients with NSCLC, which may provide a new target for the targeted therapy of NSCLC.

YB1 is a nucleic acid binding protein encoded by YBX1 gene and was initially identified as a Y‐box binding factor, which has many functions, including translation regulation, transcription regulation, stress response, DNA repair and so on.^28^, ^29^ YB1 protein is highly expressed in many kinds of malignant tumors, and studies have found that YB1 protein could bind to suppressor gene P53 and inhibit P53 promoter transcription, thereby reducing P53 activity.^30^, ^31^ It was reported by Birgit et al that YB1 expression was up‐regulated and translocated to nucleus, which was a crucial factor in proliferation, migration and chemosensitivity of melanoma cells.^31^ Besides, YB1 could positively regulate the expression of epidermal growth factor receptor (EGFR) and human epidermal growth factor receptor 2 (HER2) and promote the proliferation of breast cancer cells.^10^, ^32^ Wang et al^33^ also found a significant correlation between YB1 expression and HER2 positivity. In lung adenocarcinoma cells, Ha *et al* demonstrated that YB1 could promote the EMT mediated by transforming growth factor beta 1 (TGF‐β1) and promote the invasion and metastasis of cancer cells.^34^ What's more, it was validated that YB1 expression was higher in NPC specimens, negatively related to membrane E‐cadherin levels, but positively related to vimentin expression and associated with T stage and metastasis.^35^ These results mentioned were consistent with our findings. Our results showed that YB1 was highly expressed in NSCLC and was correlated with clinical stage, which indicated YB1 might play a key role in the development of NSCLC. Moreover, Kaplan‐Meier analysis revealed that the OS rate was significantly lower for NSCLC patients with high expression of YB1, but it was not a significant prognosis factor in multivariate analysis, which needs to be further investigated.

AKT is a 57kDa threonine/serine protein kinase, which acts as a key molecule of PI3K/AKT/mTOR pathway^11^ and p‐AKT, as a state of functional activation, is the key to its biological activity,^36^ which plays a vital role in promoting the growth, proliferation and angiogenesis of tumors, inhibiting cell apoptosis and accelerating the invasion and metastasis of tumors.^12^ It was confirmed by David et al that AKT activation was an early and frequent event in lung carcinogenesis and might increase the risk of developing a malignant tumor, which was an independent adverse prognostic factor of NSCLC.^37^ Besides, Activation of AKT signaling was confirmed in 60%‐70% of human colon cancers and abnormal activation of AKT in CRC (colorectal cancer) promoted proliferation and inhibited apoptosis of cancer cells, and was associated with depth of invasion, vascular invasion, LNM and tumor stage.^38^, ^39^ Another research revealed that p‐AKT was involved in the occurrence and development of thyroid cancer and was closely associated with the metastasis of tumor cells.^40^ It was also observed that p‐AKT induced epithelial mesenchymal transition and enhanced motility and invasiveness of squamous cell carcinoma lines.^41^ In this study, we found that p‐AKT was higher in NSCLC compared with noncancerous lung tissues, which was in accordance with the previous findings. Also, the expression of p‐AKT in lung SCC was higher than that in lung ADC, which indicated that the expression of p‐AKT correlated with histological type. In addition, the OS rate was significantly higher for NSCLC patients with low expression of p‐AKT, but there was no significant difference in multivariate analysis, which might partly make a compliment to the role of p‐AKT.

Our data also showed that G3BP1 was positively correlated with YB1 in mRNA level and the high expression of G3BP1 protein positively correlated with the expression of YB1 and p‐AKT proteins, but the association between YB1 and p‐AKT protein was not clear, which need more experiments to further confirm. It has been reported that YB‐1 could bind to the 5’UTR of G3BP1 transcripts to up‐regulate translation, thus controlling the validity of the G3BP1 SG nucleator in SG assembly and promoting the tumor progression, which might explain the positive correlation between G3BP1 and YB1, and indicated they may promote the occurrence and development of NSCLC together. Elevated expression of G3BP1 protein was also positively related to the expression of p‐AKT, which indicated the formation of SGs and G3BP1 might activate of PI3K/AKT signaling pathway and both of them might promote each other in the biological behavior of NSCLC. It has been shown that knockdown of G3BP1 downregulated the expression level of p‐PI3K (Tyr458) and p‐AKT (Ser473) in esophageal cancer cells,^42^ which supports our hypothesis that G3BP1 has a potential role in regulating PI3K/AKT singling pathway. In future research, we will do more experiments to further prove the role of G3BP1 on regulating PI3K/AKT pathway in NSCLC. In addition, in next studies, we will do some more in‐depth experiments to rule out other possibilities in regulating AKT phosphorylation, such as growth factors^43^ and Notch1.^44^

In conclusion, G3BP1 may play a crucial role in activating PI3K/AKT/mTOR signaling pathway. Overexpression of G3BP1 might be a promising independent poorer prognostic marker for NSCLC patients, which afforded new insights into the study on the mechanism and therapy of NSCLC. In order to further verify the roles of G3BP1 in NSCLC and its effect on biological behavior, more studies were needed.

## CONFLICT OF INTEREST

There are no conflicts of interest to declare.
